# Heart rate variability during auricular acupressure at the left sympathetic point on healthy volunteers: a pilot study

**DOI:** 10.3389/fnins.2023.1116154

**Published:** 2023-06-02

**Authors:** Dieu-Thuong Thi Trinh, Hoang-Linh Thi Le, Minh-Man Pham Bui, Khac-Minh Thai

**Affiliations:** ^1^Faculty of Traditional Medicine, University of Medicine and Pharmacy at Ho Chi Minh City, Ho Chi Minh City, Vietnam; ^2^Faculty of Pharmacy, University of Medicine and Pharmacy at Ho Chi Minh City, Ho Chi Minh City, Vietnam

**Keywords:** auricular acupressure, sympathetic point, heart rate variability, Vaccaria ear seed, vagus nerve

## Abstract

**Introduction:**

This research is a pilot, single-blinded study investigating heart rate variability (HRV) during auricular acupressure at the left sympathetic point (AH7) in healthy volunteers.

**Methods:**

There were 120 healthy volunteers with hemodynamic indexes (heart rate, blood pressure) within normal ranges, randomly divided into two groups AG and SG (in each group having a gender ratio 1:1, aged 20−29), to receive either auricular acupressure using ear seed (AG) or sham method using adhesive patches without seed (SG) at the left sympathetic point while lying in a supine position. Acupressure intervention lasted 25 min, and HRV was recorded by a photoplethysmography device–namely, Kyto HRM-2511B and Elite appliance.

**Results:**

Auricular acupressure at the left Sympathetic point (AG) led to a significant reduction in heart rate (HR) (*p* < 0.05) and a considerable increase in HRV parameters demonstrated by HF (High-frequency power) (*p* < 0.05), compared to sham auricular acupressure (SG). However, no significant changes in LF (Low-frequency power) and RR (Respiratory rate) (*p* > 0.05) were observed in both groups during the process.

**Conclusion:**

These findings suggest that auricular acupressure at the left sympathetic point may activate the parasympathetic nervous system while a healthy person is lying relaxed.

## 1. Introduction

Cardiovascular disease is one of the leading causes of death worldwide, accounting for 30% of all cases (Grenier et al., 2016b; Namara et al., 2019). In addition to the main risk factors (including hypertension, dyslipidemia, and smoking habits), dysregulation of the autonomic nervous system with increased sympathetic activity and decreased parasympathetic activity is an important predictor of cardiovascular diseases and vascular events such as hypertension, myocardial infarction, arrhythmia (Licht et al., 2013; Cortez et al., 2015; Grenier et al., 2016a). Cardiac autonomic dysfunction can be measured non-invasively using heart rate variability (HRV) (Hillebrand et al., 2013). Many studies have shown that chronic low HRV values are associated with sudden cardiac death while reduction of HR and increase of HF can be considered a beneficial direction for cardiovascular diseases (Haarala et al., 2011; Terziyski et al., 2016; Sessa et al., 2018).

Auricular acupressure using Vaccaria seed has become a crucial type of acupuncture widely applied in daily clinical practice. The seed of Vaccaria segetalis, known as Wang-Bu-Liu-Xing, is often used in auricular acupressure for physical stimulation and is common in Vietnam and some other countries, such as Chinese, Japan, and Korea. Vaccaria seeds are carefully selected for their size (about 2 mm in diameter), placed on adhesive patches, and packaged for clinical convenience; then, the clinical practitioner easily presses one seed at each acupoint and kneads each acupoint for a particular period. Auricular acupressure using Vaccaria ear seed is a relatively safe treatment with minimal adverse effects, e.g., itchiness of the ear which is a tolerable side effect. Evidence for auricular acupuncture’s therapeutic effects comes from clinical practice and research into pain control, headaches, schizophrenia, and depression; it also helps effectively treat back pain and primary dysmenorrhea of females and opiate addiction (Bearn et al., 2009; Zhao et al., 2011; Hou et al., 2015; Baker and Chang, 2016; Cha and Sok, 2016; Jonas et al., 2016; Zhao and Ma, 2018). The current evidence suggests that auricular acupuncture is closely related to the autonomic nervous system (He et al., 2012). More specifically, auricular acupuncture is performed on acupoints in the distribution area of the vagus nerve of the concha, increasing parasympathetic activity (Thayer et al., 2010). The shift toward parasympathetic dominance contributes to an increment of HRV, suggesting a potentially beneficial effect on cardiovascular function (Marlow, 2014). Sympathetic acupoint (AH_7_), located in the area innervated by ear branch of the vagus nerve (ABVN), is scientifically proven to affect the autonomic nervous system and is widely used to treat many diseases relating to autonomic nervous system disorders (Young and McCarthy, 1998; Kreuzer et al., 2012; Cha and Sok, 2016; De Lorent et al., 2016). Studies have also suggested that vagus nerve stimulation should often be performed on the left side to avoid cardiac complications because the right vagus nerve strongly influences the sinus node, which can cause cardiac arrest (Kreuzer et al., 2012).

We found limited research on the effect of stimulating only the sympathetic point and using auricular acupressure, a method with little risk and many benefits. Therefore, this research was conducted to evaluate the effect of auricular acupressure at the left Sympathetic acupoint (AH7) on the autonomic nervous system by using an HRV measuring device in healthy individuals. In this study, the values of features of the frequency domain of HRV (low-frequency power - LF, high-frequency power - HF), heart rate–HR, and respiratory rate–RR were obtained to identify if there are any significant changes during the process of auricular acupressure [from the time the adhesive patches with/without ear seed is glued (T3) to the time it is removed (T8)]. At the same time, we surveyed undesirable events (pain due to pressure, skin irritation, nausea, syncope) after applying the intervention.

This study result can support the effectiveness of auricular acupressure in HRV improvement and suggest this method as an additional treatment for ANS dysfunction-related diseases in future research based on its impact on parasympathetic stimulation and inhibition of sympathetic activity.

## 2. Materials and methods

The study was designed as a single-blinded pilot study. Participants were healthy volunteers who lived in Ho Chi Minh City. The Medical Ethics Council of the University of Medicine and Pharmacy at Ho Chi Minh City approved the research ethics. Volunteers would sign an informed consent form before the study. Participants were randomly assigned into two groups by the GraphPad software version 9.1. Participants in the auricular acupuncture group received auricular acupressure in the left Sympathetic acupoint, while the control group received sham acupressure by removing the ear seed but keeping the sticker attached in such an acupoint. The participants do not know whether they are in the sham acupressure.

The sample size was calculated according to the formula.


$$\text{n}\geq \frac{{\left(z_{1-\beta }+z_{1-\alpha /2}\right)}^{2}.\sigma^{2}}{d^{2}}$$

Where n is the number of sample sizes needed for the study; z1-β = 0.83, z1- α/2 = 1.96. According to Clancy et al. (2014) *d* = 70, 35, σ = 178.65. With a 10% expected loss, n must be more than 57; for this reason, each group’s sample size is 60, with two groups totaling 120 people.

### 2.1. Inclusion criteria

Healthy males and females aged between 20 and 29 with no history of cardiovascular diseases, diabetes, or thyroid, and had vital signs within the normal range (pulse, regular heart rate, resting heart rate: 60−100 beats/min, resting blood pressure: from 90 to 60 mmHg to ≤140/90 mmHg, breathing rate: 16 ± 3 times/min, temperature: 36.6−37.5 C), body mass index (BMI) from 18.5 to 23 kg/m^2^, had no psychiatric stress problem during acupuncture day (confirmed by answering the DASS21 questionnaire with stress point less than 15 points).

### 2.2. Exclusion criteria

Volunteers whose ages were out of the range above used stimulants such as beer, alcohol, coffee, and tobacco within 24 h before conducting the study. No volunteers played sports 2 h before participating in the research or; had skin injuries in the area of auricular acupressure. Women in menstruation, pregnancy, breastfeeding and people using drugs affecting blood pressure and heart rate within 1 month were also excluded.

### 2.3. Criteria to stop research

Participants who wanted to stop participating in the study or had overreacted parasympathetic stimulation symptoms such as dizziness, nausea, vomiting, pain, and allergy at the stimulus area. These cases would be recorded as unexpected events.

### 2.4. HRV measuring

Monitoring values in the periods included before (T1 and T2), during (T3, T4, T5, T6, and T7), and after (T8) auricular acupressure using the photoplethysmography device–namely, Kyto HRM-2511B (from Kyto Electronic Co., Limited on China Suppliers)–attached to the left earlobe of participants and connected with the Elite HRV App of the mobile phone. The results showed that with HRV studies measured at rest, the photoplethysmography (PPG) device correlated with ECG from 0.85 to 0.99. Monitored values included heart rate (HR) and HRV, measured by frequency-domain features (LF and HF).

The HRV measurement standards were developed by the European Society of Cardiology (ESC) and the North American Society of Cardiac Rhythm and Electrophysiology (NASPE) in 1996 and have become a popular measurement standard. The frequency domain of HRV (LF and HF components) is commonly applied in studies on HRV, and assessing the cardiac autonomic nervous system is possible through these HRV components.

LF (low-frequency power–LF has a frequency of 0.04–0.15 Hz): has a relationship with cardiac sympathetic activity.

HF (high-frequency power–HF has a frequency of 0.15–0.4 Hz): reflects cardiac parasympathetic modulation.

### 2.5. Auricular acupressure

We conducted auricular acupressure at the Sympathetic acupoint on the left ear, which was located in the middle of the ear cavity, by using a sticker with Vaccaria ear seed (auricular acupressure group–AG) or sticker without seed (sham group–SG) for 25 min with 3 times of stimulating. The stimulation time was 30 s with 2 acupressure movements per second, resulting in 60 acupressure movements per stimulation. HRV was monitored every 5 min before and after stimulating sessions during this stage (T3, T4, T5, T6, and T7). The acupuncturist was trained at the University of Medicine and Pharmacy of Ho Chi Minh City and had 5 years of experience in performing auricular acupressure.

### 2.6. The final stage (after acupressure)

**FIGURE 1:**
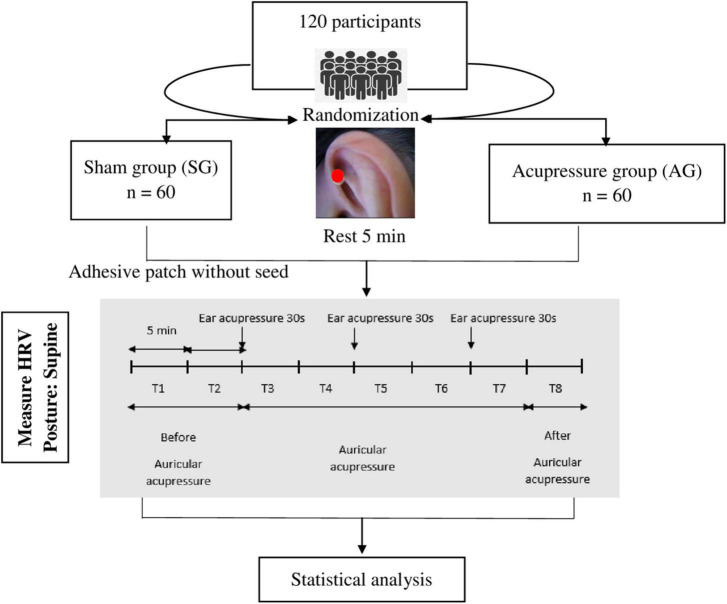
Study protocol. T1, T2: Before auricular acupressure, T3: The 1^st^ auricular acupressure with stimulation in 30 s, T4: Auricular acupressure without stimulation, T5: The 2^nd^ auricular acupressure with stimulation in 30 s, T6: Auricular acupressure without stimulation, T7: The 3^rd^ auricular acupressure with stimulation in 30 s, T8: After auricular acupressure.

All subjects underwent a 5-min HRV measurement after removing adhesive patches with or without seed (T8). The measurement profile and times (T1-T8) are shown schematically in Figure 1.

### 2.7. General protocol

The study was conducted in a quiet room from 8:00 to 10:00 A.M. at 26 ± 1°C. Participants rested for 10 min, and then their HR, RR, and blood pressure were measured. Participants did not speak and did not change their posture during acupressure.

The total number of participants was 120, and we arranged for 2−3 people to participate in the study each day.

The recording duration was 5 min/stage, and the total time was 40 min.

A typical PPG wave has peaks and valleys representing blood volume changes in peripheral tissue (earlobe) due to blood pulses. PPG pulses can therefore be associated with ECG R peaks, they are delayed because of transit time through blood vessels, and pulse-to-pulse (PP) intervals can be used as a reliable replacement for RR intervals in many applications. However, this model Kyto HRM-2511B does not provide information about sampling frequency.

Using OMRON HEM 7121 arm blood pressure monitor to measure participants’ blood pressure. The researcher observed respiratory rates in each stage (T1, T2, T3, T4, T5, T6, T7, and T8) and measured by counting the number of times the participant’s abdomen rise-down.

### 2.8. Statistical analysis

Data were analyzed using SPSS version 22.0. Independent Samples *T*-test was used to compare baseline characteristics of the volunteers between two groups for each stage. We used the Wilcoxon signed rank test to compare HR, RR, LF, and HF at time points (before, during, and after acupressure) in each group and the Mann-Whitney *U*-test for differences in these parameters between the two groups. The results were statistically significant when *p* < 0.05.

If the participants had any symptoms such as dizziness, nausea, vomiting, pain, and allergy at the stimulus area, the study would be stopped and recorded as unexpected events.

## 3. Results

There were no participants discontinued or excluded from the trial. All 120 participants were recorded, and their data were transferred to the blinded analyst. It took 55 consecutive minutes to finish the process for one person and 40 days for 120 participants.

### 3.1. Heart rate (HR) in each stage of the study

**FIGURE 2:**
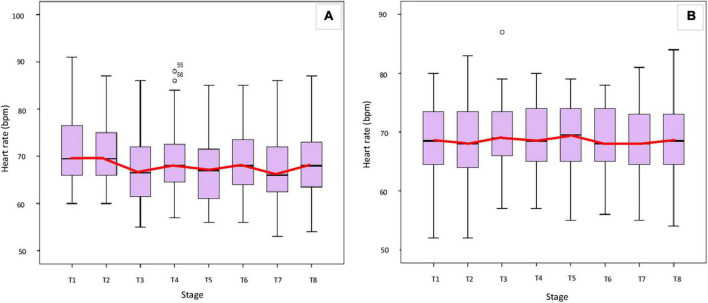
Heart rate at each stage in the two groups. **(A)** AG, **(B)** SG.

Figure 2 shows the HR of each stage in the two groups, and there were no statistically significant differences in HR at each stage T1, T2, T3, T4, T5, T6, T7, and T8 between the two study groups (*p* > 0.05 Mann-Whitney *U*-test).

Besides, there was a statistically significant difference in HR between stages T3, T4, T5, T6, T7, and T8 with stages T1 and T2 between the two groups (*p* < 0.05 Mann-Whitney *U*-test). Specifically, in the acupressure group (AG), HR in the stage of auricular acupressure with stimulation was lower than that of before, after, and in the stage without stimulation (Figure 2A) (*p* < 0.05, Wilcoxon signed rank sum test). In contrast, the HR differences between stages in the sham group (SG) were not statistically significant (Figure 2B) (*p* > 0.05, Wilcoxon signed rank sum test).

### 3.2. Auricular acupressure at Sympathetic acupoint alters elements of HRV and RR

The variation of frequency-domain elements normalized (nLF and nHF) and the respiratory rate (RR) is shown in Table 1. There were some minor changes in nLF and nHF indicated by the light blue color in Table 2; however, these changes are discrete and lack significance.

**TABLE 1:** The change of nLF, nHF, and RR during stages in both groups.

Stage	nLF [mean (SD)]			nHF [mean (SD)]			RR [median (IQR)]		
	**AG *n* = 60**	**SG *n* = 60**	***P*-value^b^**	**AG *n* = 60**	**SG *n* = 60**	***P*-value^b^**	**AG *n* = 60**	**SG *n* = 60**	***P*-value^a^**
T1	6.18 (1.08)	5.87 (1.15)	0.14	6.61 (0.99)	6.71 (0.91)	0.57	16.00 (13.00, 18.00)	16.00 (14.00, 17.00)	0.81
T2	6.17 (1.11)	5.80 (1.28)	0.09	6.65 (0.98)	6.77 (0.88)	0.51	16.00 (13.25, 18.00)	16.00 (14.00, 17.75)	0.78
T3	6.29 (1.19)	5.92 (1.15)	0.09	6.92 (1.14)	6.75 (0.98)	0.38	16.00 (13.00, 19.00)	16.00 (14.00, 17.00)	0.50
T4	6.22 (1.20)	5.86 (1.10)	0.09	6.85 (1.06)	6.69 (0.98)	0.38	16.00 (14.00, 18.00)	16.00 (14.00, 18.00)	0.89
T5	6.27 (1.22)	5.93 (1.11)	0.11	6.91 (1.13)	6.68 (0.93)	0.23	16.00 (14.00, 18.00)	16.00 (13.00, 18.00)	0.46
T6	6.39 (0.98)	6.05 (1.03)	0.07	7.03 (1.14)	6.71 (1.02)	0.10	16.00 (14.00, 18.00)	16.00 (13.25, 17.00)	0.58
T7	6.38 (1.16)	5.91 (1.22)	0.03	6.95 (1.09)	6.73 (1.06)	0.27	16.00 (14.00, 18.00)	16.00 (14.00, 17.75)	0.82
T8	6.27 (0.95)	5.97 (1.10)	0.11	7.03 (1.07)	6.74 (3.65)	0.13	16.00 (14.00, 17.00)	16.00 (13.25, 17.00)	0.99

^a^Mann Whitney *U*-test, ^b^*t*-test; RR, respiratory rate; IQR, interquartile range.

**TABLE 2:** The difference among stages within each group and between two groups.

Stage	Compared with	nLF			nHF			RR		
		***P*-value^c^**		***P*-value^d^**	***P*-value^c^**		***P*-value^d^**	***P*-value^b^**		***P*-value^a^**
		**AG *n* = 60**	**SG *n* = 60**		**AG *n* = 60**	**SG *n* = 60**		**AG *n* = 60**	**SG *n* = 60**	
T1	T2	0.85	0.37	0.41	0.41	0.23	0.86	0.37	0.67	0.31
	T3	0.46	0.67	0.77	<0.01	0.63	0.04	0.81	0.13	0.32
	T4	0.72	0.98	0.79	0.03	0.83	0.05	0.88	0.74	0.98
	T5	0.54	0.67	0.86	0.01	0.76	0.03	0.99	0.76	0.62
	T6	0.08	0.04	0.81	<0.01	0.99	<0.01	0.72	0.32	0.62
	T7	0.17	0.80	0.45	<0.01	0.81	0.04	0.95	0.90	0.95
	T8	0.31	0.28	0.99	<0.01	0.71	<0.01	0.49	0.92	0.93
T2	T3	0.47	0.42	0.98	<0.01	0.81	0.02	0.53	0.25	0.18
	T4	0.71	0.68	0.95	0.04	0.38	0.03	0.30	0.95	0.58
	T5	0.52	0.42	0.90	0.02	0.28	0.01	0.39	0.34	0.22
	T6	0.08	0.03	0.86	<0.01	0.50	<0.01	0.82	0.17	0.21
	T7	0.17	0.47	0.64	0.02	0.69	0.03	0.57	0.51	0.27
	T8	0.31	0.10	0.64	<0.01	0.72	<0.01	0.94	0.52	0.40
T3	T4	0.63	0.74	0.98	0.48	0.23	0.95	0.83	0.31	0.49
	T5	0.92	0.98	0.94	0.95	0.18	0.60	0.82	0.95	0.84
	T6	0.49	0.32	0.91	0.26	0.31	0.16	0.42	0.59	0.71
	T7	0.50	0.92	0.57	0.80	0.66	0.70	0.76	0.55	0.71
	T8	0.91	0.74	0.75	0.25	0.84	0.26	0.34	0.99	0.57
T4	T5	0.72	0.69	0.94	0.54	0.91	0.55	0.80	0.11	0.29
	T6	0.19	0.17	0.93	0.04	0.71	0.09	0.22	0.07	0.73
	T7	0.32	0.80	0.65	0.35	0.41	0.63	0.67	0.52	0.88
	T8	0.66	0.45	0.77	0.02	0.13	0.11	0.13	0.28	0.89
T5	T6	0.31	0.37	0.99	0.13	0.54	0.26	0.25	0.45	0.60
	T7	0.46	0.93	0.59	0.72	0.34	0.92	0.47	0.45	0.20
	T8	0.99	0.75	0.83	0.20	0.26	0.53	0.20	0.80	0.25
T6	T7	0.93	0.36	0.48	0.34	0.39	0.25	0.63	0.09	0.44
	T8	0.23	0.45	0.79	0.98	0.46	0.72	0.90	0.15	0.27
T7	T8	0.39	0.68	0.38	0.38	0.86	0.48	0.55	0.67	0.73

^a^Mann Whitney *U*-test, ^b^Wilcoxon signed rank sum test, ^c^paired *t*-test, ^d^*T*-test; LF, low-frequency power; HF, high-frequency power; RR, respiratory rate.

Following the details in Tables 1, 2, there were no significant differences in the nLF, nHF, and RR between the two groups in each stage (*t*-test and Mann-Whitney *U*-test, *p* > 0.05). However, there was a considerable increase in the nHF of the acupressure group (AG) in the period during and after intervention (T3, T4, T5, T6, T7, and T8), while the sham group (SG) had no changes with the Paired *t*-test used.

Besides, Tables 1, 2 illustrate that the acupressure group (AG) had a significant increase in nHF of stages T3, T4, T5, T6, T7, and T8 compared to T1 and T2 (*p* < 0.05, Paired *t*-test); and there were no statistically significant differences in nHF between the stages T3, T4, T5, T6, T7, and T8 (*p* > 0.05, Paired *t*-test). However, these tables show the sham group (SG) had no statistically significant differences in nHF between the stages T1, T2, T3, T4, T5, T6, T7, and T8 (*p* > 0.05, Paired *t*-test). The comparison of the research results to prior studies is showed in Table 3.

**TABLE 3:** Comparison the results of our study and prior researches.

Research	HR	RR	HF	LF	Adverse effects
Gao X.Y.	Acupressure stimulation of the vagus nerve in the ear causes a reduction in heart rate			LF increased in both periods of acupoint stimulation with electromagnetism (electric vibrating pen) and vibration of needles, which differs from our study using pressure on seeds (less strong physical stimulation)	
Boehmer A.A.					
Buschman H.P.	The timing of stimulation does not affect the amount of cardiac slowing				
Kuehl L.K.		The respiratory rate must be considered to ensure a thorough interpretation of frequency-domain indices	Reduced HRV indicates a risk of cardiac and all-cause mortality; low HF is often correlated with stress status and anxiety disorders, whereas increased HF and HRV are related to wellbeing		
Laborde S.					
Tran N.		Left ear stimulation increased RSA and significantly increased HF			
Haker E.		The measuring HF and LF method was calculated appropriately to control the respiratory system’s impact on these indices	An increase in HF and no change in LF during auricular acupuncture in the left Lung acupoint		
Clancy A.J.				Performing vagus nerve acupuncture and acupressure in the ear showed no change in LF	
Boehmer A.A.					
Caetano J.	Heart rate decreases will lead to a significant decrease in both cardiovascular-related and all-cause mortality				
Khan H.					
Clancy A.J.					No undesirable events when vagus nerve stimulation
Roger A.S.					
Our study	Heart rate markedly declined during and after acupressure at the left Sympathetic acupoint in healthy volunteers, and stages with stimulation caused a more significant decrease than without stimulation. No resonance to increase an effect or saturation to decrease such impact in subsequent stimuli	Respiratory rate while evaluating HRV showed no significant change, thus excluding the effect of the respiratory system on HF	HF increased statistically significantly when performing acupressure	No statistically significant differences in LF among study periods in both groups	No undesirable events

Almost our results are similar to the prior research, except for the study of Gao X.Y. in which the LF increased when acupoint stimulation using an electric vibrating pen and vibration of needles. Differences with other research may be due to the difference in the action method, and the discussion of this part was in detail.

## 4. Discussion

### 4.1. Heart rate (HR)

The first value to be investigated when performing cardiac acupressure in the ears was the heart rate (HR). AG saw a significant decline (*p* < 0.05) in HR during the stages of acupressure compared with before-intervention. In particular, the three stages of auricular acupressure with stimulation (T3, T5, and T7) caused a more significant decrease in HR (*p* < 0.05) than the stage of non-stimulation (T4 and T6). These differences in AG were statistically significant compared with SG (*p* < 0.05). These results are similar to that of several previous studies by Gao et al. (2012) and Boehmer et al. (2020). on healthy volunteers that acupressure stimulation of the vagus nerve in the ear causes a reduction of heart rate. Our results also show no statistically significant difference among the first, second, and third stimuli, demonstrating no resonance to increase an effect or saturation to decrease such impact in subsequent stimuli. This result is similar to results from Buschman et al. (2006) which showed that the timing of stimulation does not affect the amount of cardiac slowing. In his research, the effect of stimulation carried out at 2 min intervals in pigs remains unchanged when the stimulus is applied quickly after the results of a preceding stimulus have resided. Additionally, this research showed that performing auricular acupressure on Sympathetic acupoint with stimulation probably reduced heart rate. Other studies have shown that heart rate decreases will lead to a significant decrease in both cardiovascular-related and all-cause mortality rates (Caetano and Delgado, 2015; Khan et al., 2015). For this reason, our research outcome can be used for its application in daily clinical practice to normalize the autonomic imbalance (especially sympathetic dominance) corresponding to daily changes and treat patients with cardiovascular diseases relating to autonomic dysfunction.

### 4.2. Respiratory rate (RR)

With the short-term recordings, distinct but overlapping mechanisms generate HRV measurement. Several mechanisms involve regulatory processes that control heart rate through respiratory sinus arrhythmias RSA, baroreceptor reflexes (negative feedback mechanisms controlled by blood pressure), and rhythmic changes in vascular tone. Respiratory sinus arrhythmia (RSA) refers to an increase or decrease in heart rate with respiration through the vagus nerve. Because longer expirations allow more acetylcholine metabolism, slower breathing rates can produce greater RSA, lower heart rate, and increase the value of HRV variables involving HF (Shaffer et al., 2014). Therefore, the authors Kuehl et al. (2015) and Laborde et al. (2017) suggested that when researching to investigate HRV, especially HF, to assess the parasympathetic function of the heart, it is necessary to monitor the respiratory parameters.

In this study, respiratory rate showed no significant change (*p* > 0.05) at any time point in both groups, thus excluding the effect of the respiratory system on examining HRV during auricular acupressure intervention.

### 4.3. The frequency-domain spectral analysis

#### 4.3.1. HF and nHF

Theoretically, stimulating the acupuncture point in the ear of the vagus nerve will increase parasympathetic activity, and HF, modulated by parasympathetic activity, can increase. This finding is compatible with our outcome; in particular, HF (raw and normalized values) increased dramatically during and after acupressure compared to before (*p* < 0.05), and there were no statistically significant differences between the stage with stimulation and that with non-stimulation (*p* > 0.05) in the acupressure group. However, there were no differences at any time in the sham group.

Hayano and Yuda (2019) suggested that HF is only affected by parasympathetic activity in the heart in the frequency range of 0.15−0.4 Hz, and RSA is the determining factor in HF. The effects of respiratory parameters on RSA act independently of the level of cardiac parasympathetic activity. Tran et al.’s (2019) study showed that left ear stimulation increased RSA and significantly increased HF. Kuehl et al. (2015) and Laborde et al. (2017) suggested that the respiratory rate must be considered to ensure a thorough interpretation of frequency-domain indices.

Besides, our results showed that HF (raw and normalized values) increased statistically significantly when performing acupressure. This result is similar to the study reported by Haker et al. (2000) which showed an increase in HF and no change in LF during auricular acupuncture in the left Lung acupoint. This acupoint is located in the distribution of the vagus nerve, similar to the Sympathetic acupoint in our study. A special feature in Haker et al.’s (2000) study is that the measuring HF and LF method was calculated appropriately to control the respiratory system’s impact on HF and LF. In our research, monitoring simultaneous respiratory rate while evaluating HRV showed no significant change, thus excluding the effect of the respiratory system on HF.

Reduced HRV indicates a risk of cardiac and all-cause mortality; low HF is often correlated with stress status and anxiety disorders, whereas increased HF and HRV are related to wellbeing (Kuehl et al., 2015). Our study showed that auricular acupressure at the left Sympathetic acupoint increased HF, raising the question of whether this benefit would happen in stressed subjects and then can improve their body health.

#### 4.3.2. LF and nLF

In general, there were no statistically significant differences in LF (raw and normalized values) among study periods (*p* > 0.05) intragroup and intergroup in our study. This result is similar to the studies of Clancy et al. (2014) and Boehmer et al. (2020) when performing vagus nerve acupuncture and acupressure in the ear but different from the study of Gao et al. (2012). In Gao et al.’s (2012) research, LF increased in both periods of acupoint stimulation with electromagnetism (electric vibrating pen) and vibration of needles, which differs from our study using pressure on seeds (less strong physical stimulation). In addition, the hemodynamic change when stimulating the vagus branch depends on the number of stimulation sites and stimulation parameters (potential, frequency, pulse length, and current direction), leading to varying degrees of HRV, accounting for the variation in results between studies (Stauss, 2017).

In our study, the result generally pointed out that auricular acupressure at the left Sympathetic acupoint with the Vaccaria ear seed did not change the LF (raw and normalized values). The difference with other studies may be due to the difference in the action method.

#### 4.3.3. Unwanted reactions

When acupressure at the left Sympathetic acupoint, both acupressure and sham groups did not record any undesirable events, such as pain in acupressure, skin irritation, nausea, or syncope. This result is similar to other studies using auricular acupressure on points in the distribution area of the vagus nerve (Clancy et al., 2014; Roger et al., 2016).

#### 4.3.4. Limitations

It is the first study to conduct acupressure at the left Sympathetic acupoint to investigate HRV; hence we performed it on healthy volunteers to ensure safety and to monitor possible dangerous cardiovascular events during acupressure. Evaluating results on healthy people will not represent the goal of clinical application with subjects who are cardiovascular patients. Therefore, our further research will be focused on patients with chronic cardiovascular or HRV-related diseases. The sampling frequency of the device Kyto HRM-2511B is unknown, which is considered an unclear assessment quality. However, as stated above, the Kyto HRM-2511B has been studied as a possible substitute for ECG in the short-term measuring and monitoring HRV parameters.

## 5. Conclusion

The research results reveal that heart rate markedly declined, and HF significantly increased during and after acupressure at the left Sympathetic acupoint in healthy volunteers, specifically compared to the period before performing the acupressure. When we consider the respiratory rate monitoring while performing HRV measurement to warrant more accuracy, it shows no considerable change at any time point and no significant difference between the two groups. There is no risky event recorded throughout this trial.

## Data availability statement

The original contributions presented in this study are included in the article/supplementary material, further inquiries can be directed to the corresponding authors.

## Ethics statement

This study was conducted according to the guidelines of the Declaration of Helsinki and approved by the Institutional Review Board (or Ethics Committee) of the University of Medicine and Pharmacy at Ho Chi Minh City on 28 September 2020, with the registration number: 605/HÐÐÐ-ÐHYD. Informed consent was obtained from all subjects involved in the study.

## Author contributions

D-TT and K-MT: conceptualization, methodology, and supervision. H-LL and M-MB: data curation, formal analysis, and investigation. D-TT: funding acquisition. D-TT, M-MB, and K-MT: project administration, resources, and validation. H-LL and K-MT: software. D-TT, H-LL, and M-MB: visualization. D-TT, H-LL, M-MB, and K-MT: writing—original draft and writing—review and editing. All authors contributed to the article and approved the submitted version.

## References

[B1] Baker T. E. Chang G. (2016). The use of auricular acupuncture in opioid use disorder: a systematic literature review. *Am. J. Addict.* 25 592–602. 10.1111/ajad.12453 28051842

[B2] Bearn J. Swami A. Stewart D. (2009). Auricular acupuncture as an adjunct to opiate detoxification treatment: effects on withdrawal symptoms. *J. Subst. Abuse Treat.* 36 345–349. 10.1016/j.jsat.2008.08.002 19004596

[B3] Boehmer A. A. Georgopoulos S. Nagel J. Rostock T. Bauer A. Ehrlich J. R. (2020). Acupuncture at the auricular branch of the vagus nerve enhances heart rate variability in humans: an exploratory study. *Heart Rhythm.* 1 215–221. 10.1016/j.hroo.2020.06.001 34113874 PMC8183808

[B4] Buschman H. P. Storm C. J. Duncker D. J. (2006). Heart rate control via vagus nerve stimulation. *Neuro. Technol. Neur. Int.* 9 214–220. 10.1111/j.1525-1403.2006.00062.x 22151709

[B5] Caetano J. Delgado A. J. (2015). Heart rate and cardiovascular protection. *Eur. J. Int. Med.* 26 217–222. 10.1016/j.ejim.2015.02.009 25704330

[B6] Cha N. H. Sok S. R. (2016). Effects of auricular acupressure therapy on primary dysmenorrhea for female high school students in South Korea. *J. Nurs. Scholar.* 48 508–516. 10.1111/jnu.12238 27541067

[B7] Clancy J. A. Mary D. A. Witte K. K. (2014). Non-invasive vagus nerve stimulation in healthy humans reduces sympathetic nerve activity. *Brain Stimulat.* 7 871–877. 10.1016/j.brs.2014.07.031 25164906

[B8] Cortez M. M. Nagi R. S. K. Goodman B. (2015). Autonomic symptom burden is associated with MS-related fatigue and quality of life. *Multi. Scler. Relat. Dis.* 4 258–263. 10.1016/j.msard.2015.03.007 26008943

[B9] De Lorent L. Agorastos A. Yassouridis A. Kellner M. Muhtz C. (2016). Auricular acupuncture versus progressive muscle relaxation in patients with anxiety disorders or major depressive disorder: a prospective parallel group clinical trial. *J. Acupunct. Merid. Stud.* 9 191–199. 10.1016/j.jams.2016.03.008 27555224

[B10] Gao X. Y. Wang L. Gaischek I. Michenthaler Y. Zhu B. Litscher G. (2012). Brain-modulated effects of auricular acupressure on the regulation of autonomic function in healthy volunteers. *Evid. Based Comple. Alternat. Med.* 1 1–8. 10.1155/2012/714391 21904563 PMC3163402

[B11] Grenier A. Brassard P. Bertrand O. F. (2016a). Rosiglitazone influences adipose tissue distribution without deleterious impact on heart rate variability in coronary heart disease patients with type 2 diabetes. *Clin. Auton. Res.* 26 407–414. 27498095 10.1007/s10286-016-0373-7

[B12] Grenier A. Brassard P. Bertrand O. F. Despres J. P. Costerousse O. Almeras N. (2016b). Rosiglitazone influences adipose tissue distribution without deleterious impact on heart rate variability in coronary heart disease patients with type 2 diabetes. *Clin. Auton. Res.* 6 407–414. 27498095 10.1007/s10286-016-0373-7

[B13] Haarala A. Kahonen M. Eklund C. (2011). Heart rate variability is independently associated with C-reactive protein but not with Serum amyloid A. The cardiovascular risk in young finns study. *Eur. J. Clin. Invest.* 41 951–957. 10.1111/j.1365-2362.2011.02485.x 21323913

[B14] Haker E. Egekvist H. Bjerring P. (2000). Effect of sensory stimulation (acupuncture) on sympathetic and parasympathetic activities in healthy subjects. *J. Auton Nerv. Syst.* 79 52–59. 10.1016/s0165-1838(99)00090-9 10683506

[B15] Hayano J. Yuda E. (2019). Pitfalls of assessment of autonomic function by heart rate variability. *J. Physiol. Anthropol.* 38 2–8. 10.1186/s40101-019-0193-2 30867063 PMC6416928

[B16] He W. Wang X. Shi H. Shang H. Li L. Jing X. (2012). Auricular acupuncture and vagal regulation. *Evid. Based Comple. Alternat. Med.* 78 30–39. 10.1155/2012/786839 PMC3523683 23304215

[B17] Hillebrand S. Gast B. Mutsert R. Swenne A. Jukema J. W. Middeldorp S. (2013). Heart rate variability and first cardiovascular event in populations without known cardiovascular disease: meta-analysis and dose–response meta-regression. *Eur. J. Pacing Arrhyth. Card. Electrophysiol.* 5 742–749. 10.1093/europace/eus341 23370966

[B18] Hou P.-W. Hsu H.-C. Lin Y.-W. Tang N.-Y. Cheng C.-Y. Hsieh C.-L. (2015). The history. Mechanism, and clinical application of auricular therapy in traditional Chinese medicine. *Evid. Based Comple. Alternat. Med.* 49 56–84. 10.1155/2015/495684 PMC4707384 26823672

[B19] Jonas W. B. Bellanti D. M. Paat C. F. (2016). A randomized exploratory study to evaluate two acupuncture methods for the treatment of headaches associated with traumatic brain injury. *Med. Acupunct.* 28 113–130. 10.1089/acu.2016.1183 27458496 PMC4926228

[B20] Khan H. Kunutsor S. Kalogeropoulos A. P. (2015). Resting heart rate and risk of incident heart failure: three prospective cohort studies and a systematic meta-analysis. *J. Am. Heart Assoc.* 4 13–64. 10.1161/JAHA.114.001364 25589535 PMC4330063

[B21] Kreuzer P. M. Landgrebe M. Husser O. Resch M. (2012). Transcutaneous vagus nerve stimulation: retrospective assessment of cardiac safety in a pilot study. *Front. Psychiatry* 2:70–71. 10.3389/fpsyt.2012.00070 22891061 PMC3413045

[B22] Kuehl L. K. Deuter C. E. Richter S. Schulz A. Rüddel H. Schächinger H. (2015). Two separable mechanisms are responsible for mental stress effects on high frequency heart rate variability: an intra-individual approach in a healthy and a diabetic sample. *Int. J. Psychophysiol.* 95 299–303. 10.1016/j.ijpsycho.2014.12.003 25500224

[B23] Laborde S. Mosley E. Thayer J. F. (2017). Heart rate variability and cardiac vagal tone in psychophysiological research - recommendations for experiment planning, data analysis, and data reporting. *Front. Psychol.* 8:213. 10.3389/fpsyg.2017.00213 28265249 PMC5316555

[B24] Licht C. M. De Geus E. J. Penninx B. W. (2013). Dysregulation of the autonomic nervous system predicts the development of the metabolic syndrome. *J. Clin. Endocrinol. Metab.* 98 2484–2493. 10.1210/jc.2012-3104 23553857

[B25] Marlow A.-M. (2014). Auriculotherapy Manual: chinese and western systems of ear acupuncture. *Acupunct. Med. J. Br. Med. Acupunct. Soc.* 32 294–295. 10.1136/acupmed-2014-010591

[B26] Namara K. Alzubaidi H. Jackson J. K. (2019). Cardiovascular disease as a leading cause of death: how are pharmacists getting involved. *Int. Pharm. Res. Pract.* 8 1–11. 10.2147/IPRP.S133088 30788283 PMC6366352

[B27] Roger A. S. Jane M. G. Cynthia D. C. (2016). Pilot evaluation of auricular acupressure in end-stage lung cancer patients. *J. Palliat. Med.* 19 556–558. 10.1089/jpm.2015.0347 26835562

[B28] Sessa F. Anna V. Messina G. (2018). Heart rate variability as predictive factor for sudden cardiac death. *Aging* 10 166–177. 29476045 10.18632/aging.101386 PMC5842851

[B29] Shaffer F. McCraty R. Zerr C. L. (2014). A healthy heart is not a metronome: an integrative review of the heart’s anatomy and heart rate variability. *Front. Psychol.* 5:1040. 10.3389/fpsyg.2014.01040 25324790 PMC4179748

[B30] Stauss H. M. (2017). Differential hemodynamic and respiratory responses to right and left cervical vagal nerve stimulation in rats. *Physiol. Rep.* 5 21–24. 10.14814/phy2.13244 28400500 PMC5392529

[B31] Terziyski K. V. Draganova A. I. Taralov Z. Z. (2016). The effect of continuous positive airway pressure on heart rate variability during the night in patients with chronic heart failure and central sleep apnoea. *Clin. Exp. Pharmacol. Physiol.* 43 1185–1190. 10.1111/1440-1681.12662 27560005

[B32] Thayer J. F. Yamamoto S. S. Brosschot J. F. (2010). The relationship of autonomic imbalance, heart rate variability and cardiovascular disease risk factors. *Int. J. Cardiol.* 141 122–131. 10.1016/j.ijcard.2009.09.543 19910061

[B33] Tran N. Asad Z. Elkholey K. (2019). Autonomic neuromodulation acutely ameliorates left ventricular strain in humans. *J. Cardiovasc. Trans. Res.* 12 221–230. 10.1007/s12265-018-9853-6 30560316 PMC6579714

[B34] Young M. F. McCarthy P. W. (1998). Effect of acupuncture stimulation of the auricular sympathetic point on evoked sudomotor response. *J. Alternat. Comple. Med.* 4 29–38. 10.1089/acm.1998.4.1-29 9553833

[B35] Zhao J. Q. Ma T. M. (2018). A meta-analysis on acupuncture and moxibustion treatment of schizophrenia. *Zhen Ci Yan Jiu.* 43 806–812. 30585461 10.13702/j.1000-0607.170716

[B36] Zhao L. Guo Y. Wang W. (2011). Systematic review on randomized controlled clinical trials of acupuncture therapy for neurovascular headache. *Chin J. Integr. Med.* 17 580–586. 10.1007/s11655-011-0709-z 21526365

